# Supplementary material for: Laquinimod treatment attenuates EAU by inhibiting both the inductive and effector phases in an APC-dependent manner
Source: bioRxiv. 2025 May 25:2025.05.20.654165. Preprint. [Version 1] doi: 10.1101/2025.05.20.654165 (PMC12148052; doi:10.1101/2025.05.20.654165)
Supplement: Supplement 1 [file NIHPP2025.05.20.654165v1-supplement-1.pdf]

bioRxiv preprint doi: https://doi.org/10.1101/2025.05.20.654165; this version posted May 25, 2025. The copyright holder for this preprint (which was not certified by peer review) is the author/funder. This article is a US Government work. It is not subject to copyright under 17 USC 105 and is also made available for use under a CC0 license.

Supplementary Figures

bioRxiv preprint doi: https://doi.org/10.1101/2025.05.20.654165; this version posted May 25, 2025. The copyright holder for this preprint (which was not certified by peer review) is the author/funder. This article is a US Government work. It is not subject to copyright under 17 USC 105 and is also made available for use under a CC0 license.

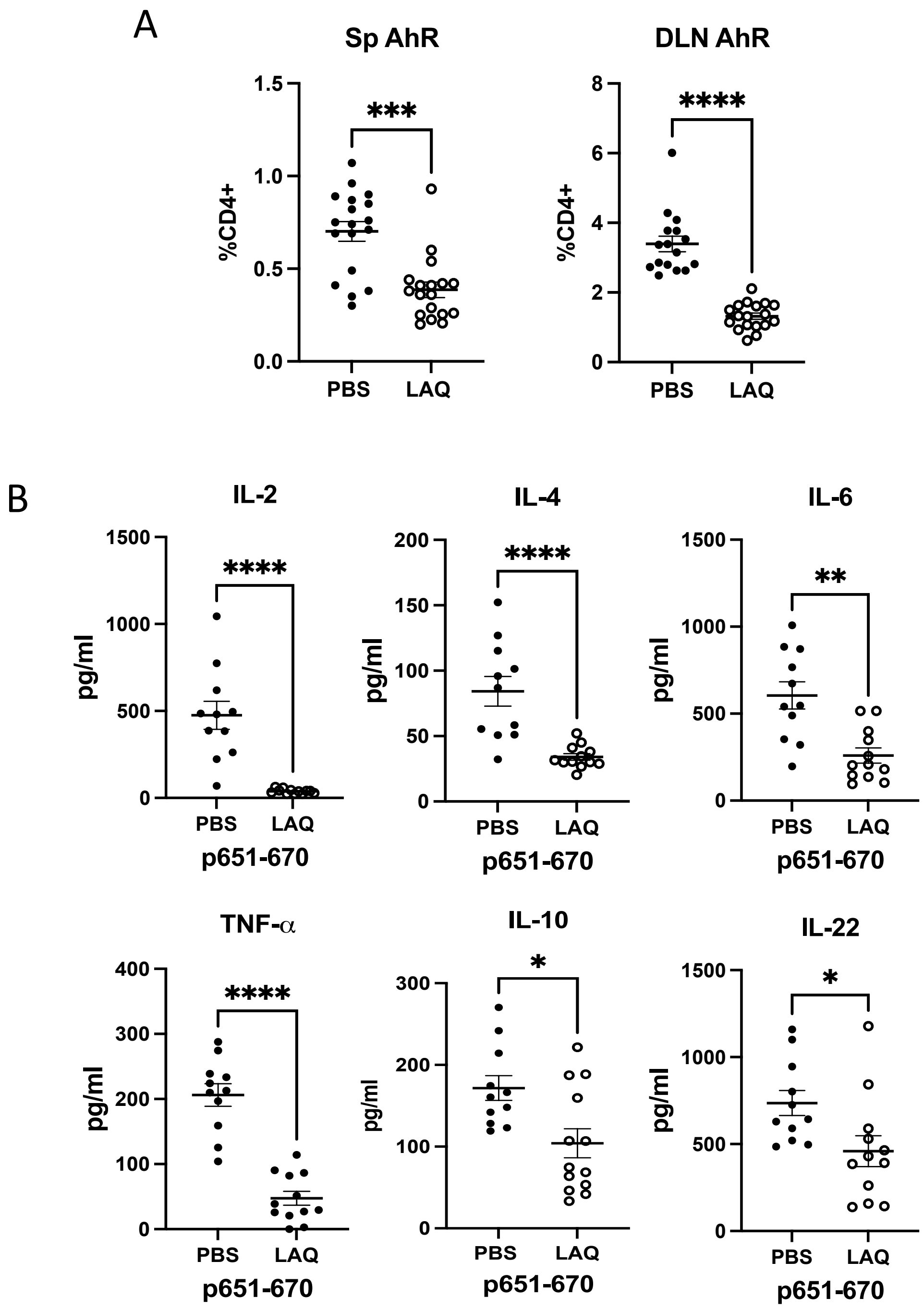

Figure S1. LAQ reduces AhR⁺ cells and broadly suppresses cytokine responses

EAU mice were euthanized on day 21 post-immunization. Cells from the spleen (SP) and draining lymph nodes (DLN) were processed for ex vivo testing. **A -** Intracellular AhR expression levels were tested by flow cytometry without stimulation. **B -** IL-2, IL-4, IL-6, TNF-α, IL-10 and IL-22 ELISA on culture supernatants of splenocytes stimulated with p651-670. Each dot represents one mouse, and all data were plotted as mean ± SEM. The Mann-Whitney test was used to determine the significance. *p <0.05, ** p <0.01, *** p <0.001, ****p <0.0001.

bioRxiv preprint doi: https://doi.org/10.1101/2025.05.20.654165; this version posted May 25, 2025. The copyright holder for this preprint (which was not certified by peer review) is the author/funder. This article is a US Government work. It is not subject to copyright under 17 USC 105 and is also made available for use under a CC0 license.

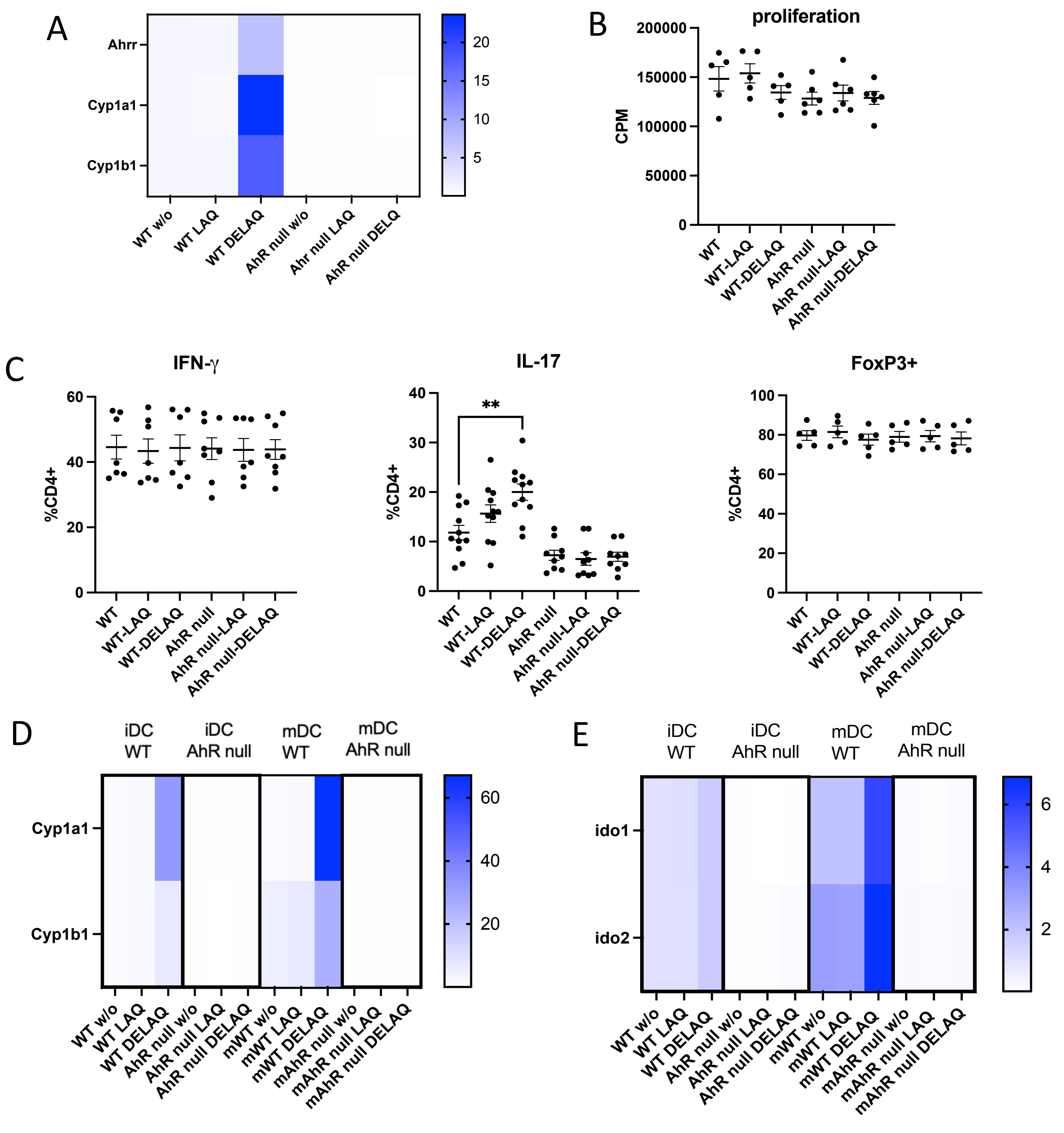

Figure S2: DELAQ activity is AhR-dependent and APC-mediated

A–C: Naïve CD4+ T cells, purified from WT or AhR null mice, were stimulated with plate-bound anti-CD3 and anti-CD28 antibodies. Cells were treated with LAQ or DELAQ at 100ng/ml for 24 hours. **A** – q-PCR analysis on AhR pathway-related genes, *Ahrr, Cyp1a1,* and *Cyp1b1*. RQ values were plotted. **B -** Proliferative response to LAQ or DELAQ treatment. **C -** cells were polarized to Th1, Th17, and Treg with cytokine cocktails and analyzed by flow cytometry. **D & E**: BMDC generated from B6 and AhR null mice were treated with LAQ or DELAQ at 100 ng/ml for 24 hours. **D -** q-PCR analysis on AhR pathway-related genes *Cyp1a1* and *Cyp1b1*. Combined data from three experiments. **E -** q-PCR analysis on *Ido1* and *Ido2* gene expression. Combined data from three experiments. Each dot represents one experiment. All data were plotted as mean ± SEM. The Mann-Whitney test was used to determine the significance. *p <0.05, **p <0.01, ***p <0.001.
